# Noncontact evaluation of full elastic constants of perovskite MAPbBr_3_ via Photoacoustic eigen-spectrum analysis in one test

**DOI:** 10.1038/s41598-020-66938-2

**Published:** 2020-06-19

**Authors:** Tao Zhang, Xiao-yu Liu, Chao Tao, Xiangxing Xu, Xiao-jun Liu

**Affiliations:** 1 MOE Key Laboratory of Modern Acoustics, Department of Physics, Collaborative Innovation Center of Advanced Microstructures, Nanjing University, Nanjing, 210093 China; 2 School of Chemistry and Materials Science, Nanjing Normal University, Nanjing, 210046 China; 3 Shenzhen Research Institute of Nanjing University, Shenzhen, 518000 China

**Keywords:** Applied physics, Condensed-matter physics, Techniques and instrumentation

## Abstract

Elasticity is one basic property of hybrid organic-inorganic perovskites. It highly relates to many fundamental processes in solid physics. The investigation of elasticity is of interest not only to explore the intrinsic properties of a material, but also to improve their potential application performance. In this study, we predict photoacoustic eigen-spectrum (PAES) of single crystal. Then by solving the inverse problem of the generation of PAES, we propose a noncontact method to determine a complete set of elastic constants of single crystal in one test. Experiments confirm the proposed method accurately determines all elastic constants of MAPbBr_3_. Since this method is totally noncontact and does not require multiple specimens cutting along different crystal axes, it could be more competent for rare, tiny and brittle specimen, or when the specimen is immersed in turbid or opaque medium. Benefitting from these superiorities, the proposed method might be found prominent values in materials science and applications.

## Introduction

Hybrid organic-inorganic perovskites (HOIPs) are one of the most promising photovoltaic materials, benefitting from their high charge carrier motilities, low temperature solution process, long charge carrier diffusion lengths, superb optical absorption across the visible spectrum, and high photoelectric conversion efficiency^1–5^. Elasticity is one important property of HOIPs, which is associated with many fundamental processes in solid physics, including lattice dynamics, phonon spectra, thermal expansion, Debye temperature and Grüneisen constant^6^. Therefore, the investigation of elasticity is of interest not only to explore the intrinsic properties of a material, but also to improve their potential application performance^7–10^.

Many works have been done to predict or measure the elasticity of HOIPs. The first principle was used to theoretically calculate the elastic constants according to the equation for the interatomic potential^7^. Inelastic neutron scattering spectroscopy (INS) and Brillouin light scattering (BS)^11,12^ have been applied to measure the elasticity of HOIPs. These methods detect the scanning scattering energy, extract the speed of sound in different directions, then derivate the elastic constants. Whereas, these methods often need multiple specimens and to cut samples in different directions. Resonant ultrasonic spectroscopy (RUS) was also applied to evaluate the elasticity of HOIPs with a direct-contact excitation of the eigen-vibrations of the specimen^8,13–18^. Non-contact resonant ultrasonic spectroscopy techniques use pulse laser to excite vibrations, and detect the surface displacement of the sample by using optical method^19–21^. This method could be restricted by the surface condition and shape of the specimen, and environmental medium. Whereas, a more applicable method is still demanded for exploring the elasticity of perovskites.

In this study, we report a noncontact method, to evaluate all elastic constants of a cubic phase crystal, based on the photoacoustic eigen-spectrum (PAES). Based on photoacoustic effect there are powerful technologies for imaging in opaque medium, which open up great prospects for biomedicine^22–27^. Our previous study revealed that photoacoustic signals from isotropic spherical and cylindrical elastomer contain the eigen-vibration information^28,29^. PAES are the components in photoacoustic signals corresponding to the eigen-vibration of the elastomer. In this study, we solve the forward problem of predicting PAES according to the elastic constants, and demonstrate its general numerical solution of a single crystal. Then, we study the inverse problem and evaluate the elastic constants from the measured eigen-spectrum. Finally, the method is applied to measure all elastic constants of HOIPs.

## Results

### Extraction of photoacoustic eigen-spectrum

**Figure 1:**
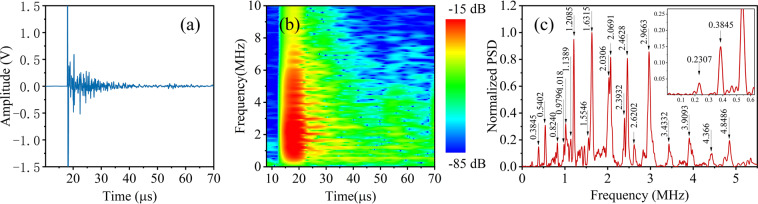
Waveform and spectrum of the photoacoustic signals. (**a**) The waveform of a photoacoustic signal. (**b**) Time-frequency spectrum of the same signal. (**c**) Normalized power spectrum density of the signal.

Figure 1 shows one typical waveform and spectrum of the photoacoustic signals detected from the specimen No. 2. As shown in Fig. 1, strong signals are generated from the specimen under the illumination of a pulse laser. Although the laser pulse is extremely narrow (8~10 ns), the ultrasound emission from the specimen last a long time (>50 µs). For the long-duration coda wave, its waveform is periodic and its amplitude is attenuated. The amplitude attenuation results from the ultrasound irradiation, which brings vibratory energy away. The periodicity corresponds to the eigen-vibration modes of the specimen. These characteristics of the signal can be confirmed in the time-frequency map, as show in Fig. 1(b). A lot of spectral lines appear in the time-frequency spectrum of the photoacoustic signals, which imply the periodic vibration of the specimen. Each spectral line corresponds to one eigen-vibration. Figure 1(c) plots the normalized power spectral density of the signals. The frequencies of eigen-vibrations can be precisely extracted from these narrow spectral peaks.

### Elastic constants derived from PAES

**Figure 2:**
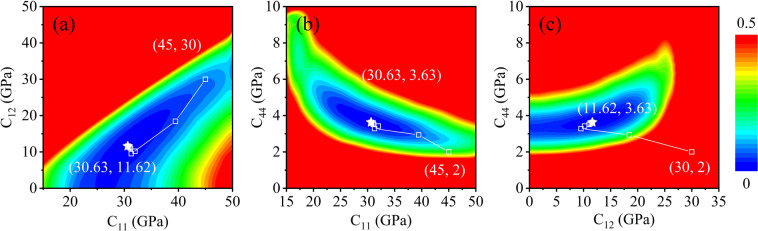
Convergence path of the inversion. (**a**) Deviation functions in *C*_*11*_-*C*_*12*_ space (**b**) Deviation functions in *C*_*11*_-*C*_*44*_ space (**c**) Deviation functions in *C*_*12*_-*C*_*44*_ space.

The elastic constants can be estimated from the measured eigenfrequencies by solving the inverse problem. Figure 2 shows the convergence procedure of elastic constants estimation, where Fig. 2(a–c) are the deviation functions in *C*_*11*_-*C*_*12*_ space, *C*_*11*_-*C*_*44*_ space, and *C*_*12*_-*C*_*44*_ space, respectively. Here, the deviation function is defined in Eq. (2). The measured frequencies *f*i^*m*^ are extracted from the photoacoustic spectrum as shown in Fig. 1. The predicted eigenfrequencies ${f}_{i}^{c}$(**x**) are calculated by solving Eq. (1) with the elastic constants given by the *x*-axis and *y*-axis. The color represents the frequency offset between the prediction values and the measured frequencies. The initial values to predict the eigenfrequencies are set as **x** = [*C*_*11*_, *C*_*12*_, *C*_*44*_] = [45 GPa, 30 GPa, 2 GPa]. Initially, the predicted frequencies are far away from the measured frequencies *F*(**x**_0_) = 0.63414, since the preset parameters do not match the accurate elastic constants of the specimen. Then, we optimize the elastic constants by solving the inverse problem. The empty dots in Fig. 2 show the convergence process of approaching the best-fitting parameters. After 10 times of iterations, the parameters are converged at **x**_opt_ = [*C*_*11*_, *C*_*12*_, *C*_*44*_] = [30.63 GPa, 11.6 GPa, 3.63 GPa] and the deviation function is reduced to *F*(**x**_opt_) = 0.001. It is seen that the parameters arrive at the valley of the deviation function, as shown by the stars in Fig. 2. The predicted frequencies match the measurements best. The optimized parameters **x**_opt_ measure the elastic constants of the specimen.

**Figure 3:**
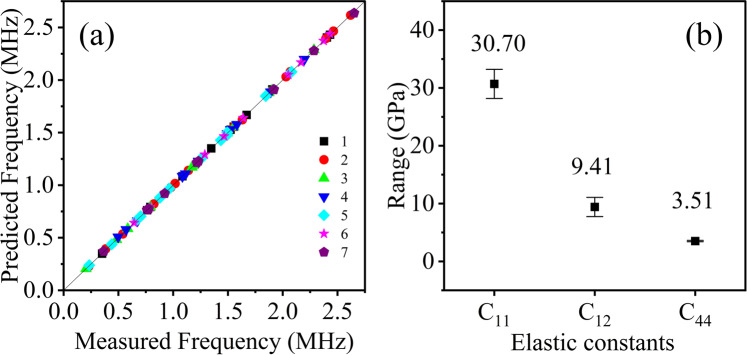
Results of the elastic measurements. (**a**) Predicted frequencies versus measured frequencies for 7 specimens, where the predicted frequencies are calculated using the optimal elastic constants. (**b**) Measured elastic constants.

Figure 3 illustrates the results of elasticity evaluation. Figure 3(a) shows the measured frequencies ${f}_{i}^{m}$ against the predicted eigen-frequencies ${f}_{i}^{c}$(**x**_opt_) for seven specimens. All frequencies agree with each other very well after solving the inverse problem. The optimized parameters **x**_opt_ can accurately predict the eigen-vibration of specimens, in other words, **x**_opt_ precisely measure elasticity of specimens. Figure 3(b) presents the measured elastic constants *C*_*11*_ = 30.7 ± 2.46 GPa, *C*_*12*_ = 10.53 ± 1.62 GPa, and *C*_*44*_ = 3.51 ± 0.11 GPa of MAPbBr_3_, and error bars indicate standard deviation of multiple measurements. The results agree with measurements by laser ultrasonic(LU), INS and BS method, reported in the previous studies, as shown in Table 1 ^11,12,30^. Moreover, the measurements of seven specimens have good consistency. The proposed noncontact method measures all elastic constants of MAPbBr_3_.

**Table 1:** Summary of elastic constants of MAPbBr_3_ measured by laser ultrasonic(LU), Brillouin light scattering(BS), inelastic neutron scattering(INS) and photoacoustic eigen-spectrum(PAES).

Elastic Constants	LU	BS	INS	PAES
C_11_ (Gpa)	32.23	32.2	34.5	30.7
C_12_ (Gpa)	9.0	9.1	18.5	10.53
C_44_ (Gpa)	3.48	3.4	4.1	3.51

## Discussion

In summary, we have predicted the PAES of a single crystal. Then by solving the inverse problem of the generation of PAES, we proposed a noncontact method to determine a full set of elastic constants of MAPbBr_3_ from the detected PAES in one test. In comparison to other methods used for the measurement of elastic constants, PAES has its own unique advantages. Firstly, PAES is totally noncontact, whether the vibration generation or the signal detection. The specimen is only simply immersed in liquid. In contact RUS system, the specimen need to be clamped by transducers with an additional force. Therefore, PAES could be applicable for brittle materials and tiny specimen. Secondly, it can probe all elastic constants of the specimen in one test. INS and BS method require multiple specimens cutting along different crystal axes. These merits make PAES method be more competent for rare specimen. Thirdly, PAES is also different from laser ultrasound that it detects the photoacoustic wave emitted from the vibrating specimen, instead of directly measuring the vibrational surface of the specimen. This method is still available when the specimen is immersed in turbid or opaque medium. Benefitting from these characteristics (or superiorities), PAES might be found valuable in some practical applications, including a precise evaluation of elasticity of tiny rare specimen, online monitoring the elastic constants during the growth process of a crystal in solutions, and testing elasticity of materials implanted in biological tissue, etc. There are also some limitations of PAES. Firstly, soft elastomer could have very low eigen-frequencies, which may be difficult to excite its vibration and detect PA eigen-spectral signals. Secondly, if specimen has big damping factor, eigen vibrations will attenuate quickly and few frequencies could be extracted. Therefore, a high quality factor of the specimen is required. Finally, the proposed method is operated in liquid, a suitable medium is required to avoid dissolution or other chemical reactions between the specimen and medium. To extract elastic constants precisely, we can enhance intensity of PA signals by increasing laser fluence and choosing laser wavelength with high light absorption. Besides, an accurate measurement of size is always important when irregularly shaped specimen is tested. Along with improving technical details, this method would be applied to more materials.

## Methods

### Measurement system

**Figure 4:**
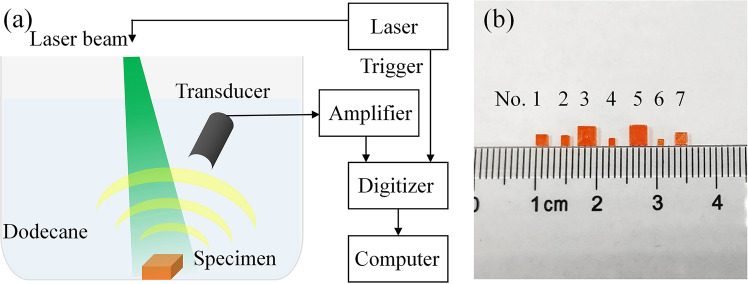
Schematic diagram of PAES experiment. (**a**) The experimental setup. (**b**) Photograph of specimens.

Figure 4(a) gives the experimental setup. The specimen was stored in a beaker and immersed in dodecane. Since the specimen contacts was freely placed in the beaker and no additional force was applied on them, the free boundary condition is approximately satisfied. A Q-switched Nd: YAG laser with a wavelength of 532 nm and a pulse width of about 8~10 ns was used to illuminate the specimen and generate photoacoustic signals. Light absorption of MAPbBr3 starts from about 570 nm and has strong optical absorption at 532 nm^31^. Based on rough prior knowledge of specimen, it can be estimated that the eigen-frequencies of the specimen are about from 0.2 MHz to 5 MHz. An ultrasound transducer (V310, Panametrics) with a central frequency of 4.39 MHz and a relative bandwidth of 100.1% at −6 dB was immersed into dodecane in the beaker to receive the photoacoustic signals. This transducer has enough wide bandwidth to cover the main frequency range of the PA signals of specimens. The detected signals were amplified 46 dB (SA-230F5, NF) and sampled by a data acquisition board (PCI-5105, NI) with a frequency of 30 MHz. For each specimen, the measurement was repeated 200 times and averaged to reduce noise. Figure 4(b) is the photograph of seven specimens of MAPbBr_3_ measured in the experiment which are prepared by the inverse temperature crystallization method^31,32^. Equimolar mixture of the CH_3_NH_3_Br and PbBr_2_ were dissolved in dimethylformamide (DMF) to obtain a 1 mol/L solution of CH_3_NH_3_PbBr_3_. The above solution was heated and kept at 80 °C for several hours, then large number of small crystals can be readily harvested. For the sake of convenience, they are numbered from 1 to 7. Their sizes were measured and given in Table 2. The volume of the smallest specimen (No. 6) is only 0.5735 mm^3^ and the biggest one (No. 5) is 15.9 mm^3^. Density is 3.8 g/cm^3^ used in this study.

**Table 2:** Dimensions of the seven rectangular specimens.

Dimension	1	2	3	4	5	6	7
2*d*_1_ (mm)	1.98	1.82	3.3	1.35	3.4	1.18	2.1
2*d*_2_ (mm)	1.95	1.4	3.04	1.08	2.96	1.08	2.04
2*d*_3_ (mm)	0.98	1	1.26	0.7	1.58	0.45	1.24

### Process of drawing time-frequency map

Time-frequency map could be calculated and drawn by the short-time Fourier transform: At the sample rate of 30 MHz, the window length and overlap size of Fourier transform are 360 and 350 separately which means time-moving step is 0.33 µs. The window type is Hanning. When drawing the time-frequency map, we use the timeline as horizontal coordinate and frequency as ordinate. Colors represent relative strength of the energy spectrum density.

### PAES prediction of elastomer

Let’s consider an elastic specimen exposed to a pulsed laser. The specimen would absorb optical energy, arise transient thermoelastic expansion, and emit wideband ultrasound to the surrounding media. This is the so-called photoacoustic effect. As pulsed laser illumination ceases, the elastomer will keep free-vibrating and emitting ultrasound for a period of time, because of its elasticity and inertance. In this stage, the frequencies of free vibrations are the eigenfrequencies of the elastomer. Moreover, the emitted ultrasound has the same frequencies as the vibrations of the elastomer. Therefore, the PAES can be predicted by analyzing eigen-vibrations of the elastomer.

Considering an elastomer with free boundaries, its eigen vibration obeys the following matrix eigenvalue equation^33^

1 $$\rho {(2\pi f)}^{2}{\bf{E}}{\bf{a}}={\boldsymbol{\Gamma }}{\bf{a}}$$

where *ρ* is the density, the components of the vector **a** = [*a*_*iλ*_] are eigen vector consists of the expansion coefficients of displacement *u*_*i*_ = *∑a*_*iλ*_*Φ*_*λ*_ which is expanded in a suitable set of basis functions *Φ*_*λ*_, and the eigenvalues correspond to the eigen frequencies *f*. **E** and **Γ** are matrices whose elements are *E*_*λiλ*′*i*′_ = *δ*_*ii*′_*∫Φ*_*λ*_*Φ*_*λ*′_*dV*, *Γ*_*λiλ*′*i*′_ = *C*_*iji*′*j*′_*∫Φ*_*λ*,*j*_*Φ*_*λ*′,*j*′_*dV* respectively. *C*_*iji*′*j*′_ is the elastic tensor and subscript indices following a comma denotes differentiation with respect to that coordinate. The forward problem of PAES generation can be solved from Eq. (1), that is, the PAES of elastomer can be predicted with the given elastic constants.

### Inversion of elastic constants

If the PAES is detected, elastic constants could be estimated by solving the inverse problem, that is, to search a set of optimized elastic constants **x**_opt_ = [*C*_*ij*_] to minimize the deviation function^16,17^

2 $$F({\bf{x}})=\mathop{\sum }\limits_{i}^{M}\,{w}_{i}{[{f}_{i}^{c}({\bf{x}})-{f}_{i}^{m}]}^{2}$$

where ${f}_{i}^{m}$ refers to the *i*-th measured frequencies and ${f}_{i}^{c}$(**x**) are the eigen-frequencies predicted by solving Eq. (1) with the elastic constants **x** = [*C*_*ij*_]. *ω*_*i*_ = 1/${({f}_{i}^{m})}^{2}$ is the weighting factor and *M* is the number of eigenfrequencies used for solving the inverse problem. The parameters to minimize the deviation function Eq. (2) can be determined by using a Levenberg-Marquardt algorithm^34–36^

3 $${{\bf{x}}}_{n+1}={{\bf{x}}}_{n}-{({{\bf{J}}}^{{\rm{T}}}{\bf{J}}+\alpha \cdot {\rm{diag}}[{{\bf{J}}}^{{\rm{T}}}{\bf{J}}])}^{-1}{{\bf{J}}}^{{\rm{T}}}{\bf{h}},$$

**x**_*n*_ is the parameter vector after *n*-th iteration, **h**(**x**) = [*h*_*1*_(**x**) *h*_*2*_(**x**) … *h*_*M*_(**x**)]^T^ with *h*_*i*_(**x**) = [${f}_{i}^{c}({\bf{x}})-{f}_{i}^{m}$]/${f}_{i}^{m}$ is the deviation vector, **J** = *d***h**/*d***x** is Jacobian matrix, and *α* is a nonnegative damping factor. With the given initial **x**_**0**_ and *α*, we can predict eigenfrequencies and calculate the first **x**_1_ according to Eqs. (2–3). With the calculated **x**_1_ the next iteration is repeated. Finally, **x** converges at its optimized value, which makes *F*(**x**) reach the minimum value. The inverse problem is solved and **x**_opt_ is the estimated elastic constants.

### Verification of PAES

**Figure 5:**
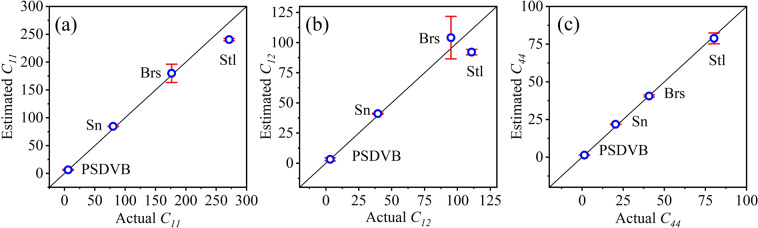
Estimated values for elastic constants (**a**) *C*_*11*_ and (**b**) *C*_*12*_ (**c**) *C*_*44*_ against their actual values.

Four spherical isotropic particles made of polystyrene divinylbenzene (PSDVB), stannum (Sn), brass (Brs), and steel (Stl) with known elasticity parameters are used to verify the accuracy of PAES. Figure 5 shows the estimated elastic constants values match the actual values very well. Zener anisotropy index A = 2*C*_*44*_/(*C*_*11*_-*C*_*12*_)^37^ of PSDVB, Sn, Brs, Stl are 0.87, 1.00, 1.07, 1.06 respectively that exhibit an elastic isotropic nature. The result confirms the accuracy of PAES method in elasticity measurement of isotropic materials.

Based on the verification of PAES, we proposed a noncontact method to measure the elasticity of the relevant member in HOIPs, named as MAPbBr_3_ that has 3 independent elastic constants **x** = [*C*_*11*_ *C*_*12*_ *C*_*44*_] in the cubic phase. A 2*d*_1_ × 2*d*_2_ × 2*d*_3_ rectangular shaped specimen is illuminated by a pulse laser and the generated photoacoustic signals are detected. Since the photoacoustic signals contain the same frequencies as the eigen-vibrations of the specimen. The elastic constants of the specimen can be evaluated by the above inverse problem.

## Data Availability

The datasets generated during and/or analyzed during the current study are available from the corresponding author on reasonable request.
